# High-efficiency focusing and imaging by dielectric kinoform zone plate lenses with soft X-rays

**DOI:** 10.1107/S1600577522012115

**Published:** 2023-02-17

**Authors:** Xujie Tong, Yifang Chen, Zijian Xu, Yijie Li, Zhenjiang Xing, Chengyang Mu, Jun Zhao, Xiangjun Zhen, Chengwen Mao, Renzhong Tai

**Affiliations:** a Nanolithography and Application Research Group, School of Information Science and Technology, Fudan University, Shanghai 200433, People’s Republic of China; b Shanghai Synchrotron Radiation Facility, Shanghai Advanced Research Institute, Chinese Academy of Sciences, Shanghai 201210, People’s Republic of China; Uppsala University, Sweden

**Keywords:** dielectric kinoform zone plate lens, greyscale electron beam lithography, high-efficiency focusing by soft X-rays, X-ray microscopy

## Abstract

With fast advances in enhancing the focusing/imaging resolution of Fresnel zone plate lenses toward sub-10 nm, low diffraction efficiency in connection with their rectangular zone shape still remains a big issue in both soft and hard X-ray microscopy. In hard X-ray optics, encouraging progress has recently been reported in our earlier attempts of high focusing efficiency by 3D kinoform shaped metallic zone plates, formed by greyscale electron beam lithography. This paper addresses our efforts towards high focusing/imaging efficiency by developing a novel dielectric kinoform zone plate lens for soft X-rays. The effects of the zone materials and zone shapes on the focusing/imaging quality were first theoretically investigated by a modified thin-grating-approximation method, revealing superior efficiencies of dielectric kinoform zone plates over rectangular ones in metals. Optical characterizations of replicated dielectric kinoform zone plates by greyscale electron beam lithography demonstrate a focusing efficiency of 15.5% with a resolution of 110 nm in the water window of X-rays. Apart from high efficiency, the novel kinoform zone plate lenses developed in this work exhibit significant advantages over conventional zone plates, *i.e.* simplified process, low cost and no need for a beamstop.

## Abstract 2

The effects of zone materials and zone shapes on the focusing/imaging quality of dielectric kinoform zone plates have been theoretically investigated by a modified thin-grating-approximation method. Greyscale electron beam lithography was applied to generate 3D kinoform zones in HSQ and PMMA for soft X-rays. The dielectric kinoform zone plate lens demonstrates high diffraction efficiency well beyond conventional X-ray optics.

## 1. Introduction

Soft X-ray microscopy is one of the most important nanoprobe techniques (Chu *et al.*, 2008 ▸; Kirz, 1974 ▸; Spector, 1997 ▸; Selin *et al.*, 2015 ▸), being widely applied in the development of, but not limited, biological science (Kapishnikov *et al.*, 2017 ▸; Dehlinger *et al.*, 2020 ▸), material science (Le *et al.*, 2020 ▸) and nanotechnology (de Smit *et al.*, 2008 ▸). Especially in the 2.3–4.4 nm wavelength range (the ‘water window’), it is particularly adequate for high-resolution imaging of biological specimens (Kepsutlu *et al.*, 2020 ▸; Chiappi *et al.*, 2016 ▸) because of the high optical contrast of the X-rays in this energy range between carbon and water present in testing materials. Good quality imaging of bio-specimens requires high photon flux for reducing the exposure time to avoid damage to cells by soft X-ray irradiation, demanding both high resolution and high focusing efficiency of the lenses.

Conventional lenses are metal-based binary Fresnel zone plates (FZPs) (Keskinbora *et al.*, 2018 ▸; Sanli *et al.*, 2018*a* ▸; Belkhou *et al.*, 2015 ▸; Chao *et al.*, 2009 ▸; Moldovan *et al.*, 2018 ▸; Mohacsi *et al.*, 2017 ▸), being widely applied in X-ray microscopes. Unfortunately, their rectangular zone shape and metal material with high absorption coefficient limits their diffractive focusing efficiency within 10% in the water window band (Chao *et al.*, 2005 ▸; Marschall *et al.*, 2017 ▸; Rösner *et al.*, 2020 ▸; Lindblom *et al.*, 2009 ▸; Reinspach *et al.*, 2011 ▸). It is understood that a zone plate with parabolic zone shape, known as a kinoform lens (Di Fabrizio, 1994 ▸), should, in principle, possess superior focusing/imaging efficiencies over rectangular lenses for both soft and hard X-rays. However, the construction of such lenses with 3D zone shape has met big challenges in both design and fabrication technique. To optimize the zone shape for maximizing efficiency, the standard beam propagation method (BPM) needs to be modified so that one can govern the focusing performance by utilizing the best possible material as well as the best designed zone structure. In the technical development, 3D lithography should be innovated for transferring the designed profile into 3D kinoform zone plate (KZP) lenses on a nanometre scale. So far, despite substantial reported attempts, significant progress at soft X-ray wavelengths has still not been seen. The development of one-dimensional (1D) kinoform lenses is inhibited by the limited aspect ratio in the deep silicon etch or LIGA technique (Fu *et al.*, 2017 ▸; Zhang *et al.*, 2016 ▸; Simons *et al.*, 2016 ▸). 3D nanoprinting has been attempted using kinoform lenses (Yu *et al.*, 2020 ▸; Sun *et al.*, 2014 ▸; Xie *et al.*, 2020 ▸) in organic materials for visible and near-infrared wavelengths, but not in the X-ray regime. Femtosecond two-photon 3D nanoprinting was applied for plastic kinoform lenses (Sanli *et al.*, 2018*b* ▸) with an efficiency of 20% in the soft X-ray region (1.5 keV) but the resolution achieved was merely 558 nm. Metal kinoform lenses based on focused ion beam (FIB) lithography (Keskinbora *et al.*, 2015 ▸) have also been attempted, resulting in 15% efficiency and 400 nm resolution at 1100 eV. The efficiency was constrained by their rough surface, and further enhancement of both the numerical aperture and the resolution would be almost impossible because of the limited writing field with FIB. Therefore, to the best of our knowledge, a kinoform zone plate lens capable of high-efficiency focusing with advanced resolution in the soft X-ray region, especially in the water window band, has still not been achieved. In the hard X-ray region, however, a trapezoid-kinoform zone plate was recently reported in our earlier work using greyscale electron beam lithography (GS-EBL) combined with gold (Au) electroplating, based on the theoretical design of the zone shape using our modified BPM method (Tong *et al.*, 2022 ▸).

In this work, inspired by our success in the hard X-ray region, a novel dielectric kinoform zone plate lens made of low-loss material, such as HSQ (hydrogen silsesquioxane) and PMMA (polymethyl methacrylate), for soft X-ray focusing/imaging with high efficiency has been developed. State-of-the-art 3D GS-EBL was applied to generate the theoretically designed kinoform shape of the zones. Using our modified BPM, the efficiencies were quantitatively compared with various lens materials including both metals and dielectrics, and an optimized kinoform zone profile suitable for GS-EBL was calculated. Optical characterizations, using a scanning X-ray microscope (STXM), of the fabricated dielectric kinoform zone plate lenses demonstrate a peak focusing efficiency of 15% in the water window band. Structural analysis of the X-ray tested kinoform zone plate lenses with HSQ using a high-resolution scanning electron microscope (SEM) shows no sign of deformation in the zone structure. The progress achieved in this work lays a solid foundation for constructing a new generation of soft X-ray lenses with both high-focusing/imaging efficiencies and high resolution.

## 2. Effects of lens structure and material on focusing efficiency

The lens structure and the material properties characterized by the refractive index, *n* = 1 − δ − *i*β, are the two most important factors determining the focusing efficiency for a phase zone plate. In the soft X-ray region, Au zone plates work mostly in binary mode because of the high absorption of light, giving rise to the theoretical limit of efficiency of 10% (Kirz, 1974 ▸). On the other hand, zone plates made of dielectric materials such as HSQ and PMMA have larger phase shifts (1 − δ) but lower absorption (β) than those made of metals, making phase modulation possible for soft X-rays. Therefore, dielectric zone plates enjoy a theoretical limit of efficiency up to 40% (Kirz, 1974 ▸), provided that the zones are both tall enough to meet the coherent diffraction on the focusing spot and robust enough to withstand X-ray radiation. Furthermore, if the dielectric zones are shaped as kinoform ones, even higher efficiency limits beyond 40% are expected (Yan, 2010 ▸; Lassaline *et al.*, 2020 ▸). Theoretical calculations of focusing efficiencies by lenses of different materials (Au, HSQ and PMMA) as well as different zone shapes (rectangular and kinoform) were carried out using our modified BPM (Tong *et al.*, 2022 ▸).

**Figure 1:**
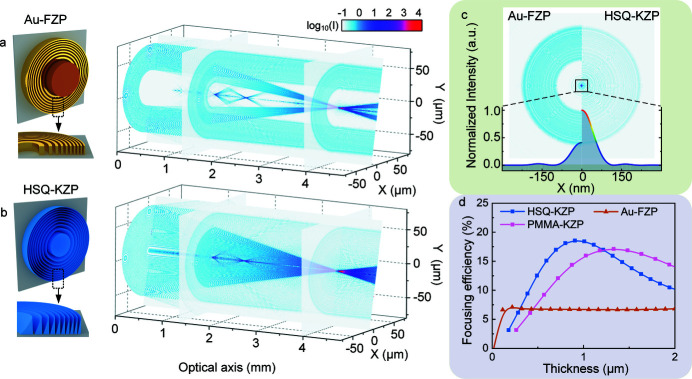
Comparisons of wavefield propagations between the HSQ kinoform zone plate (HSQ-KZP) and the metal zone plate (Au-FZP). The wavefield propagation is calculated using the beam propagation method combined with the quasi-discrete Hankel transform [BPM-QDHT (Tong *et al.*, 2022 ▸)]. The resolution of the Au-FZP (*a*) and the HSQ-KZP (*b*) is 100 nm with a diameter of 100 µm. The thickness of the Au-FZP and HSQ-KZP is 250 nm and 750 nm, respectively. The diameter of the beamstop for the FZP is 50 µm. (*c*) Comparison of point-spread functions of the focused light by both the Au-FZP and the HSQ-KZP, respectively, at the focal plane. (*d*) The calculated focusing efficiencies of the Au-FZP, HSQ-KZP and PMMA-KZP for soft X-rays (500 eV) using the BPM-QDHT approach.

X-ray propagation inside the lens was calculated using our earlier developed BPM-QDHT method (Tong *et al.*, 2022 ▸) (where QDHT stands for quasi-discrete Hankel transform). Considering that the transmitted relative light intensities and efficiencies are not influenced by the incident light intensity, the incident light field for all lenses is simplified to a plane wave with unit amplitude. The calculated wavefields propagating in 3D space through the HSQ-KZP and Au-FZP are shown in Fig. 1 ▸. In the binary FZP, as schematically illustrated in Fig. 1 ▸(*a*), the lens thickness was chosen for peak efficiency and the outermost zone width is 100 nm for both zone plates. It can be seen in Fig. 1 ▸(*a*) that the light through the centre of the binary FZP is not diffracted illumination, and must be blocked by a beamstop to ensure essential contrast. All these energy losses are responsible for the efficiency reduction in FZPs. However, by contrast, in the HSQ-KZP configuration [Fig. 1 ▸(*b*)], all the zones with the kinoform shape, including the central ones, diffract the incoming light onto the focus point, giving rise to an intensity much higher than that in the binary FZP [Fig. 1(*c*)]. Fig. 1 ▸(*d*) presents the efficiencies calculated using our BPM-QDHT method for the Au-FZP and the KZP for two different materials, HSQ and PMMA, at 500 eV. When the lens thickness reaches 250 nm, the Au-FZP collects much more light than the dielectric KZPs because its Au zone thickness satisfies the integers of the π phase shift at 500 eV for the efficiency maxima, which makes Au-FZP a phase-shifting zone plate; whereas, the same thickness for dielectric KZPs is not large enough to satisfy the integers of the π phase shift so the lenses fail to diffract most of the light onto the focus spot. When the thickness of the lens is further increased, high absorption of light disables the Au-FZPs’ ability to modulate the phase, so the lenses work in binary mode. As a result, the efficiency saturates at a low level of 6.7%, as presented by the brown curve in Fig. 1(*d*). However, on the other hand, the dielectric KZPs with low loss meet the integer multiple phase shift for coherent diffraction when the thickness increases to 1–1.5 µm and the efficiency reaches a high of 18.7%, as shown in Fig. 1(*d*), well beyond that of Au binary zone plates by a factor of two to three.

**Figure 2:**
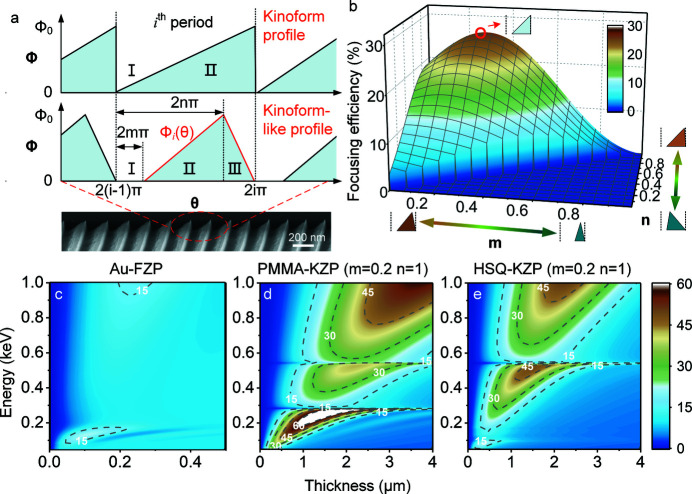
Theoretically calculated focusing efficiencies of kinoform zone plates suitable for GS-EBL for soft X-rays. (*a*) Illustrations of zone profile for KZPs. The thickness is shown in terms of the phase shift. An ideal kinoform lens has a vertical sidewall on one side; a fabricated kinoform zone plate, *e.g.* HSQ-KZP, has slopes on both sidewalls. Each zone of a real KZP is then divided into three regions with different transition function *F*(θ). *m* and *n* describe the relative position of the gap and the top of the KZP in the zone (0 < *m* < *n* < 1). (*b*) The calculated efficiency of the HSQ-KZP with thickness of 700 nm as a function of *m* and *n* at 500 eV, based on the thin grating approximation. (*c*–*e*) Theoretical calculations of the focusing efficiency of Au-FZP, PMMA-KZP (*m* = 0.2, *n* = 1) and HSQ-KZP (*m* = 0.2, *n* = 1) based on the thin grating approximation as a function of both the photon energy and the lens thickness in the soft X-ray region (0–1 keV).

It is commonly understood that the morphology of fabricated KZPs inevitably differs from the theoretically designed kinoform profile due to its complex three-dimensional topography and process limitations (Sanli *et al.*, 2018*b* ▸; Keskinbora *et al.*, 2015 ▸; Takeuchi *et al.*, 2012 ▸) such as the proximity effect (Chen, 2015 ▸) in the EBL process. To calculate the focusing/imaging efficiencies of a zone plate lens with kinoform-like morphology, a modified thin grating approximation (Kirz, 1974 ▸; Di Fabrizio & Gentili, 1999 ▸; Tatchyn *et al.*, 1984 ▸) is proposed in this work. Based on the variational formulation of zone plate theory (Tatchyn *et al.*, 1984 ▸), a more general formula for a wide energy band with varying height in zones has been developed in this work. The incident light field has wavelength λ and amplitude *C*; the number of zones is *N*. The refractive index of the material used in the optical element is expressed as *n* = 1 − δ − *i*β, where 1 − δ and β represent the real and imaginary parts of the refractive index of the material, respectively. According to the height function *t*_*i*_(θ) of the *i*th zone [Fig. 2 ▸(*a*)], the corresponding phase shift function of the *i*th zone can be expressed as

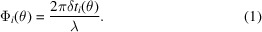

Then the amplitude *A*_*i*_ of the *i*th zone according to the thin grating approximation formula can be expressed as

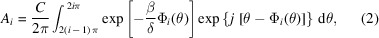

where θ is the optical path length difference to the focal spot over one ring period. Since the initial luminous flux received by each zone is the same, the total efficiency of the final optical element is the average of the efficiencies of all the wavebands. According to different height functions of the 1–*N* zones, the amplitude of each zone can be obtained. Then the overall efficiency, FE, can be expressed as

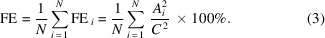

Fig. 2 ▸(*a*) presents the replicated morphology of a typical HSQ-KZP with kinoform-like profiles by GS-EBL. In order to calculate its theoretical efficiency, each zone is divided into three areas. The shallower area usually produces gaps in region I, arising from over development or insufficient exposure dose. Due to proximity effects, the sidewalls are no longer vertical in region III. The corresponding amplitudes in the three regions were calculated independently and the phase shift function can be expressed as

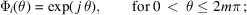




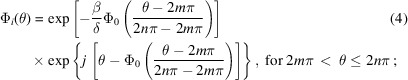




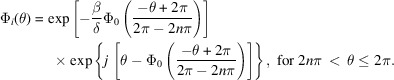

Here, structural parameters *m* and *n* describe the relative position of the gap and the top of the KZP, respectively, in one zone (0 < *m* < *n* < 1). Φ_0_ is the maximum phase shift of the lens, Φ_0_ = 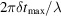 . *t*_max_ is the maximum height of the *i*th zone. Generally speaking, in the central area of the lens, *m* = 0–0.1, *n* = 0.8–0.9. In the peripheral area, *m* = 0.3–0.5, *n* = 1 (details can be found in Fig. S4 of the supporting information). We set different structural parameters *m* and *n* and repeat the efficiency calculation using equations (1) to (3). Fig. 2 ▸(*b*) presents the calculated efficiency of HSQ-KZPs of thickness 700 nm as a function of *m* and *n* at 500 eV. The lens can reach the highest efficiency when *n* = 1, *m* = 0.2, implying that the peripheral sidewall of each zone is completely vertical with a 20% gap left. The 15–25% gap in each ridge is beneficial to the efficiency improvement (Tatchyn *et al.*, 1984 ▸) and leaves a comfortable margin to tolerate the GS-EBL fabrication errors in generating 3D kinoform shapes.

In addition, it is worth noting that when the verticality of the sidewall on the outer edge of each zone becomes poor (*e.g.* *n* = 0.8), the efficiency of the KZP drops by 10%, as shown in Figs. 2 ▸(*d*) and 2(*e*). Therefore, to achieve efficient focusing, each zone of the KZP needs to have a vertical edge, which will be discussed in the fabrication part of this paper (Section 3). According to the structural parameters that can be practically realized by GS-EBL, Figs. 2 ▸(*c*), 2(*d*) and 2(*e*) present the calculated focusing efficiencies of Au-FZP, PMMA-KZP and HSQ-KZP, respectively, in the soft X-ray region under ideal *n* and *m* parameters. KZP lenses in both HSQ and PMMA demonstrate much higher focusing efficiencies than when Au is used in the water window band, although higher thickness is necessary, which is consistent with the results calculated by the BPM-QDHT method. Therefore, for soft X-rays, kinoform or kinoform-like zones with dielectric materials should be the premier choice for diffractive phase lenses with high efficiencies over metallic ones which are absorptive to X-rays.

## 3. 3D kinoform zone plate lenses by e-beam direct write

**Figure 3:**
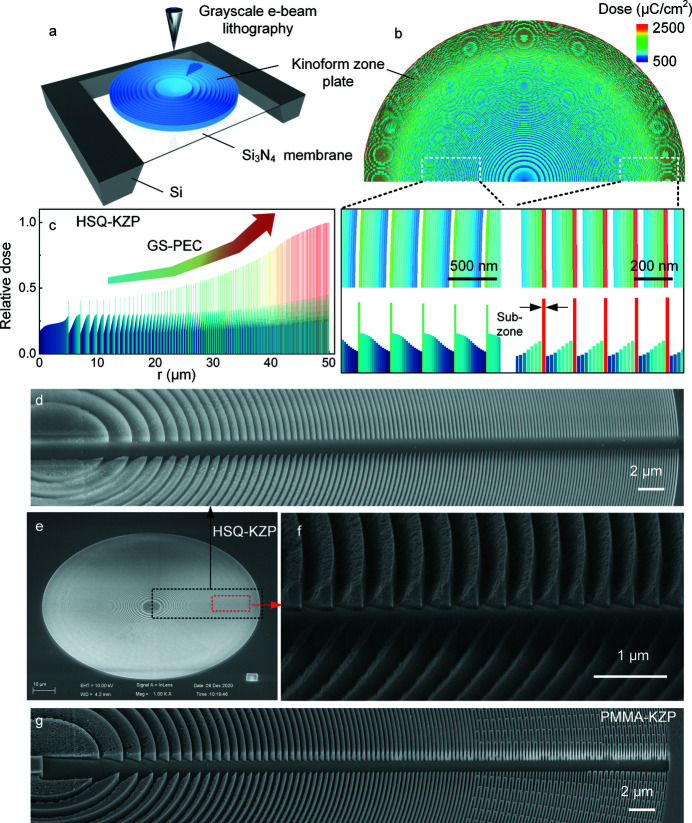
Overview of the fabrication procedures. (*a*) Schematic illustration of greyscale electron beam lithography (GS-EBL) for the HSQ-KZP lens as an example. (*b*) The calculated dose gradient pattern for the HSQ-KZP lens by GS-EBL. (*c*) Dose distribution curves for the HSQ-KZP in which the greyscale proximity effect correction (GS-PEC) was applied. (*d*) Micrographs by SEM of the cross-sectional view at a tilt of 45° for the fabricated KZP lenses in HSQ. (*e*) The whole fabricated HSQ-KZP lens. (*f*) Close-up view at a tilt of 75° of the replicated HSQ-KZP zones. (*g*) Cross-sectional view at a tilt of 45° of the PMMA-KZP lens.

To demonstrate high-efficiency focusing and imaging with soft X-rays, dielectric KZP lenses were fabricated by direct e-beam write using 3D GS-EBL in SiO_*x*_-based HSQ and PMMA, respectively. To achieve the parabolic shape for the zones in HSQ-KZP as schematically illustrated in Fig. 3 ▸(*a*), exposure dose distributions were calculated using *TRACER* and *LAB* software, delivered by GenLsys Ltd, according to the desired profiles of the lens zones.

Fig. 3 ▸(*b*) presents the calculated dose distribution by *TRACER*, seen as circles with regularly varying diameter on a 2D plane. Each zone consists of sub-zones with single pixel width of 20 nm, as shown in the inset of Fig. 3 ▸(*b*). Each sub-zone has a dosage factor ranging from 0 to 1. In order to pursue the kinoform-like topography, a large dose was assigned to the outermost circle of each zone in the hope of obtaining vertical sidewalls; meanwhile, the two innermost circles remained unexposed to obtain a gap. The overall spatial distribution of charge to be applied for generating KZP lenses is shown in Fig. 3 ▸(*c*). In order to maintain the sloping shape of the periphery and to reduce structural distortions, the greyscale proximity effect correction (GS-PEC) was also applied. By adjusting the charge distribution in the resists, the exposure dose on the outermost circle of each zone was almost doubled to obtain sharp standing triangles. Detailed exposure dose information for PMMA-KZP can be found in the supporting information.

In greyscale e-beam lithography, 750 nm-thick positive tone HSQ (Fox-15) and 1000 nm-thick PMMA (350 K) were spin-coated on 100 nm-thick SiN_*x*_ membranes, followed by a soft bake in an oven at 180°C for 1 h. E-beam exposure was carried out by using a state-of-the-art beam-writer, JBX6300 FS (see the supporting information for details). After development in TMAH:H_2_O (1:3) for HSQ and MIBK:IPA (1:3) for PMMA, respectively, HSQ- and PMMA-based 3D kinoform lenses were formed on 100 nm-thick free-standing SiN_*x*_ membranes. Figs. 3 ▸(*d*)–3(*g*) present the replicated KZP lenses with diameter of 100 µm and outermost zone-width of 100 nm in HSQ and PMMA. Accompanying KZPs with a trench across the lens centre were also prepared for inspecting the profiles of the lens zones. Clear kinoform-like profiles can be observed, especially for the HSQ-KZP lenses. Additional ribs were facilitated for the PMMA-KZP to prevent the zones from collapsing.

## 4. High efficiency focusing/imaging by kinoform zone plate lenses

**Figure 4:**
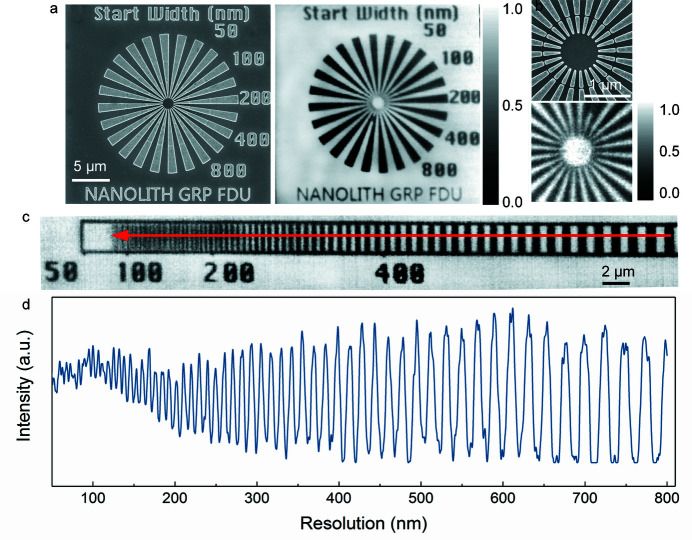
Results of soft X-ray imaging tests for the fabricated HSQ kinoform zone plate. (*a*) SEM image (left) of the in-house-made Siemens stars with resolution of 30 nm and soft X-ray image (right) of the same target at 500 eV with photon flux of 5 × 10^9^ photons s^−1^. The dwelling time is 1 ms and step size is 50 nm, using the HSQ-KZP lens as focusing component. (*b*) SEM image and soft X-ray STXM image of the magnified central part of the Siemens star, using the HSQ-KZP lens at 500 eV with dwelling time of 1 ms and 25 nm step size. The colour bars in (*a*) and (*b*) are normalized photon flux. (*c*) Image of an in-house-made grating with variable pitches, on which the red arrow line shows the scanning with a dwelling time of 2 ms and step size of 30 nm, using the HSQ-KZP lens as focusing component. (*d*) Intensity profile of the test grating over 50 pitches as outlined in (*c*) by the red arrow line.

High-efficiency focusing/imaging by the fabricated kinoform zone plate lenses was demonstrated at Shanghai Synchrotron Radiation Facility using in-house-developed STXMs with soft X-rays provided by beamline BL08U1A. Both the HSQ-KZP lens and the PMMA-KZP lens with 100 nm resolution were tested (see the supporting information for details of the optical-characterization setup). The standard testing samples, Siemens stars, were prepared in-house with a resolution of 30 nm and height of 1 µm. Fig. 4 ▸ presents TXM images of the testing samples (Siemens star) by the fabricated HSQ-KZP lenses at 500 eV. Clear images can be seen in Figs. 4 ▸(*a*) and 4 ▸(*b*). To further demonstrate the 100 nm resolution of the image, in-house-made gratings with variable pitches were scanned by the focused X-ray beam, as shown in Figs. 4 ▸(*c*) and 4(*d*). Resolved peaks with 100 nm pitch indicate the resolution capability of the HSQ-KZP lens.

**Figure 5:**
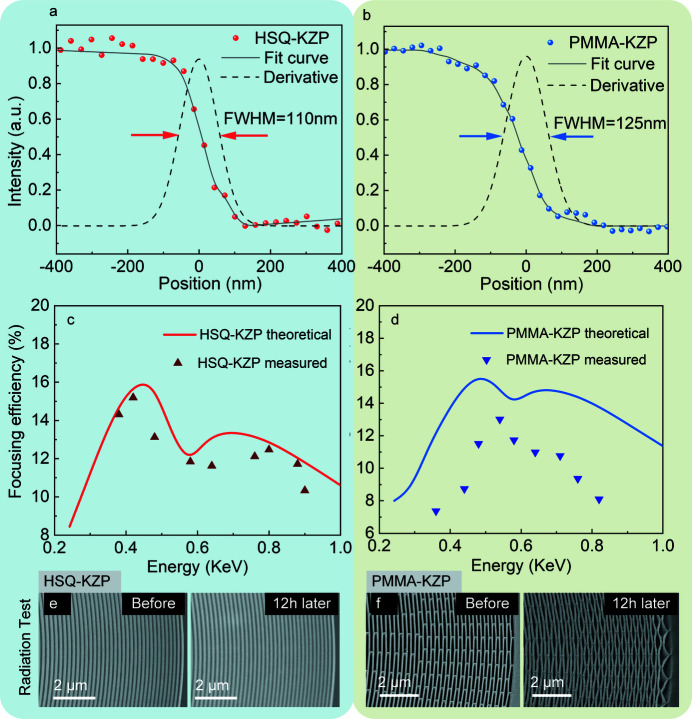
(*a*, *b*) Focusing spot profiles for soft X-rays by the HSQ-KZP and the PMMA-KZP lens measured by knife-edge scan method with dwell time of 1 ms and step size of 25 nm, where the dots are the raw data, the solid lines are the fitting curves and the dash lines are the first-order differentials of the raw data. The first-order derivatives of the scanned curves give rise to a full width at half-maximum (FWHM) of 110 nm for the HSQ-KZP lens and 125 nm for the PMMA-KZP lens. (*c*) Comparison between the measured focusing efficiencies of HSQ-KZP and the theoretical ones using the BPM-QDHT approach. (*d*) Measured focusing efficiencies of the PMMA-KZP lens compared with its theoretical value calculated by the BPM-QDHT approach. (*e*, *f*) Comparison of X-ray radiation damage to the dielectric zone plates by the SEM images of the outermost zones in the HSQ-KZP lens and the PMMA-KZP lens, after 12 h soft X-ray radiation in the tests.

Furthermore, the focusing spot dimensions of the first order were measured, using the knife-edge method, by scanning the focused spot on the line edge of the Siemen star. Figs. 5 ▸(*a*) and 5 ▸(*b*) present scanned curves using the spots focused by the HSQ-KZP and the PMMA-KZP lens, respectively. The first-order derivatives of the scanned curves give rise to a full width at half-maximum (FWHM) of 110 nm for the HSQ-KZP lens and 125 nm for the PMMA-KZP lens. Efficiencies in the energy range 0.2–1.0 keV were also measured, as shown in Figs. 5 ▸(*c*) and 5 ▸(*d*) for the HSQ-KZP lens and the PMMA-KZP lens, respectively. A peak efficiency of 15.5% at 0.45 keV was measured for the HSQ-KZP, which is about 95% of the theoretical value [Fig. 5 ▸(*c*)]. Such an efficiency enhancement by the kinoform-like profile is also repeated by the PMMA-based lens, as shown in Fig. 5 ▸(*d*). The efficiency peak of the PMMA-KZP lens is around 13.5%, which is about 80% of the theoretical value, showing a larger deviation than that of the HSQ-KZP. This is mainly caused by the structural deformation under X-ray radiation in efficiency tests, as shown in Figs. 5 ▸(*e*) and 5 ▸(*f*). After 12 h exposure to X-rays during the measurement, it was found that the ridges and the ribs collapsed in the outer area of the PMMA-based lens, indicating that PMMA-KZP was unable to survive the X-ray radiation. But no sign of X-ray radiation induced damage was observed on HSQ-KZP lenses after over 48 h exposure to X-rays for the efficiency measurement.

## 5. Conclusions

Based on our earlier success in pursuing high-efficiency focusing in hard X-ray optics by metallic kinoform zone plate lenses, this work further tackles the same issue in soft X-ray microscopy by developing dielectric kinoform zone plate lenses. Theoretical calculations using the earlier developed QDHT-BPM method prove that dielectric KZPs are able to diffract incident light in the central zones to the focal plane, which is usually blocked by a beamstop in conventional Fresnel zone plates. By extending the thin grating approximation for rectangle zones to that for 3D kinoform profiles, systematic comparisons between the dielectric KZPs and metallic FZPs show significantly enhanced focusing/imaging efficiencies of dielectric kinoform zone plates over metallic ones with rectangular shape. Greyscale electron beam lithography was successfully applied to generate the 3D kinoform zone plates in both HSQ and PMMA for the first time. As high as 15.5% efficiency with a resolution of 100 nm was achieved by the fabricated dielectric KZP lenses in the water window of X-rays, in which the central zones are used for diffracting light instead of being blocked by a beamstop. Structural inspection by a scanning electron microscope shows that the HSQ kinoform zone plate lenses are robust enough to withstand X-ray radiation. The progress made in this work opens a new avenue toward high-efficiency focusing/imaging in soft X-ray microscopy and the next step is to enhance the resolution with such a novel zone plate lens.

## 6. Related literature

The following reference, not cited in the main body of the paper, have been cited in the supporting information: Henke *et al.* (1993 ▸).

## Supplementary Material

Sections S1 to S3, Figures S1 to S4, Table S1. DOI: 10.1107/S1600577522012115/ve5163sup1.pdf
