# Supplementary material for: High-efficiency focusing and imaging by dielectric kinoform zone plate lenses with soft X-rays
Source: J Synchrotron Radiat. 2023 Feb 17;30(Pt 2):319–26. doi: 10.1107/S1600577522012115 (PMC10000800; doi:10.1107/S1600577522012115)
Supplement: Supplementary file 1 [file s-30-00319-sup1.pdf]

JOURNAL OF SYNCHROTRON RADIATION

Volume 30 (2023)

Supporting information for article:

High efficiency focusing and imaging by dielectric Kinoform zone plate lenses in soft X-rays

Xujie Tong, Yifang Chen, Zijian Xu, Yijie Li, Zhenjiang Xing, Chengyang Mu, Jun Zhao, Xiangjun Zhen, Chengwen Mao and Renzhong Tai

S1. Zone plate materials comparison

For a zone plate in a material of refractive index n=1-δ-iβ, where δ is the deviation of the real part from unity and β is the imaginary part of the index of refraction, it was found that the performance of the zone plate depends on the ratio η=β/δ(Kirz, 1974). In hard X-rays between 2 keV and 20 keV, the η value of PMMA is close to that of diamond and has small absorption, as shown in **Fig. S1(a)**. In soft X-ray band, the η of all common lens materials has increased by almost 10 times (**Fig. S1(b)**), so the absorption of the material must be the primary consideration in the design of X-ray optics. The η value of gold as a traditional zone plate material is very large, meaning that a great amount of soft x-ray will be absorbed by the lens and lost during the focusing process, which strongly deteriorates the focusing efficiency. Therefore, materials like silicon dioxide and PMMA with a small η value in water window of X-ray should be potential candidates for X-ray lenses.

S2. The process details in the fabrication of Kinoform zone plate lenses

To achieve the parabolic shape for the Kinoform zones by GS-EBL, exposure doses were assigned to the KZP lens pattern, according to the calculated exposure levels for each unit ring. Monte Carlo simulation using BEAMER/TRACER software, supplied by GenIsys Ltd, was carried out to work out the spatial distributions of the exposure dose. Proximity effect correction (PEC) by TRACER was also included throughout the simulation sequence. Developing processes of the exposed resists such as HSQ and PMMA, respectively, were simulated by the LAB simulator (GenLsys Ltd.) to figure out the resultant profiles, using the measured dissolution rates obtained from contrast curves of the particular e-beam resists. The theoretical profiles were used as a reference to guide the processing parameter adjustments. Iteration of processing parameters in the simulation was carried out to minimize the difference between the desired parabolic and the practically replicated profiles.

The optimized dose distributions and the processing flow for two different lens profiles are shown in **Fig. S2**. For the Kinoform zone plate lens in SiOx for soft X-rays, a 600-nm-thick HSQ was spin-coated on a Si_3N_4 membrane pre-coated by a seeding layer of 5 nm Cr/15 nm

Au, followed by a soft bake in oven at 180 °C for 2 min. Greyscale exposure using the dose distribution as discussed above was carried out by a beam-writer, JBX-6300 FS at 100 kV with an e-beam of 7 nm in diameter and 500 pA as the beam current. Hot developing (50°C) in TMAH: H_2O (1:3) for 2 min was applied to ensure the clearance of the residual resist. Then the sample was rinsed in de-ionized water for 30 s and dried gently with compressed nitrogen gas.

To fabricate PMMA Kinoform zone plate lenses, a 1-μm thick PMMA was first spin-coated on the 100-nm thick Si_3N_4 membrane with the seeding layer of 5 nm Cr/15 nm Au. Soft bake of the coated PMMA was done in oven at 180 °C for 1 hour. Then the 1-μm-thick resist was exposed by JEOL 6300 FS at 100 kV according to the dose distribution curve. Development in IPA:H_2O (7:3) was done at 23 °C for 4 min, then rinsed in de-ionized water for 30 seconds and finished by a dry blow with compressed nitrogen gas.

S3. Optical characterization

The optical-characterization setup is depicted in **Fig. S3**. Focusing efficiency was characterized by measuring the photocurrents with an OSA as a PIN diode. Measurements were carried out with a blank window of the chip for the incident flux (I_0); with the KF and OSA close to the focus point (I_f); with the beam blocked for the dark current (I_d). The ratio of the area of the aperture spot S_{OSA} and the area of the FZP in the shadow image (S_{lens}) were determined by the CCD camera image, with the OSA out of the optical path. The efficiency was then determined by

$$\mathrm{FE} = \frac{I_f - I_0}{\left(I_0 - I_d\right)\frac{S_{Lens}}{S_{OSA}}} \times 100\%$$

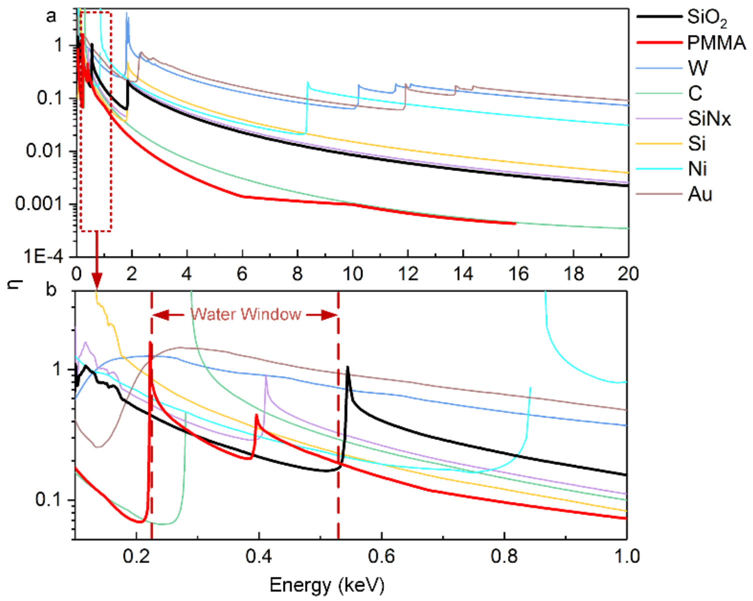

Figure S1 η for SiO_2, PMMA in hard X-ray a and soft X-ray b, compared with common X-ray optical materials. For the materials used in the calculations, the densities used are: Au: d = 19.32 g/cm^3, Ni: d = 8.90 g/cm^3; Si: d = 2.33 g/cm^3, SiNx: d = 3.44 g/cm^3, Diamond: d = 2.2 g/cm^3, W: d = 19.3 g/cm^3, PMMA: d = 1.19 g/cm^3, SiO_2: d = 2.2 g/cm^3. Data are acquired from Henke *et al.* (1993).

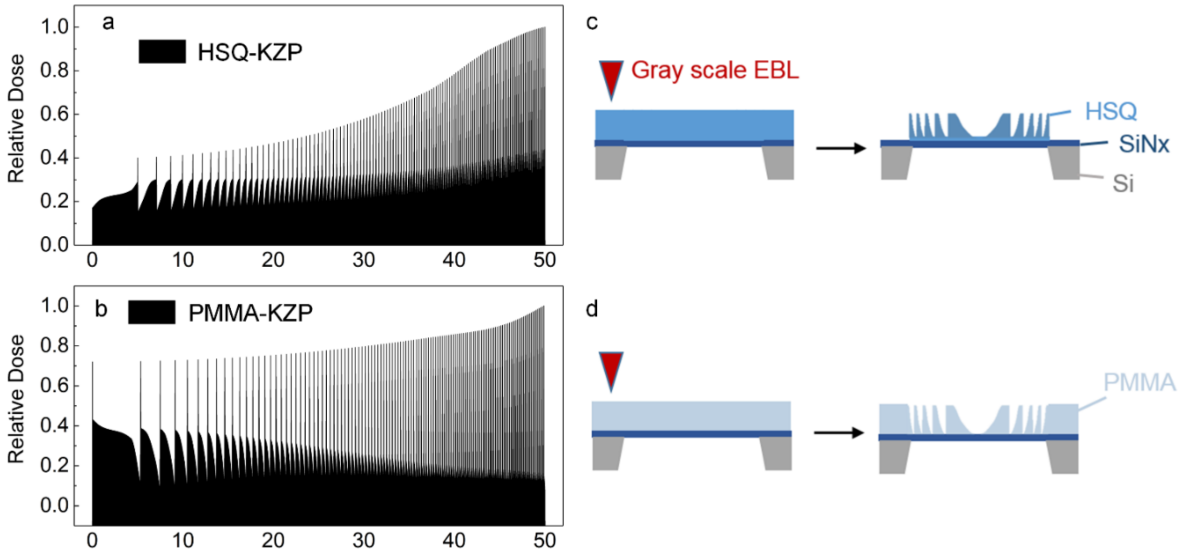

Figure S2 The optimized dose distributions for generating 3D profiles of the zones and the processing flow for three types of zone plate lenses. (a-b), the optimized dose distributions for HSQ-KZP, PMMA-KZP, respectively. (c), Illustration of the fabrication process for the HSQ-

KZP lens. Greyscale exposure using the dose distribution in a was carried out on a SiNx membrane pre-coated with a 600-nm-thick HSQ. Hot developing (50°C) in TMAH: H_2O (1:3) for 2 min was applied to clear the residual resist. The sample was then rinsed in de-ionized water for 30 s and dried gently with compressed nitrogen gas. (d), Illustration of the fabrication process of PMMA-KZP. Greyscale exposure using the dose distribution in b was carried out on a SiNx membrane which was spin-coated with a 1000-nm-thick PMMA. Development in IPA:H_2O (7:3) was done at 23°C for 4 min, then rinsed in de-ionized water for 30 seconds and finished by a dry blow with compressed nitrogen gas.

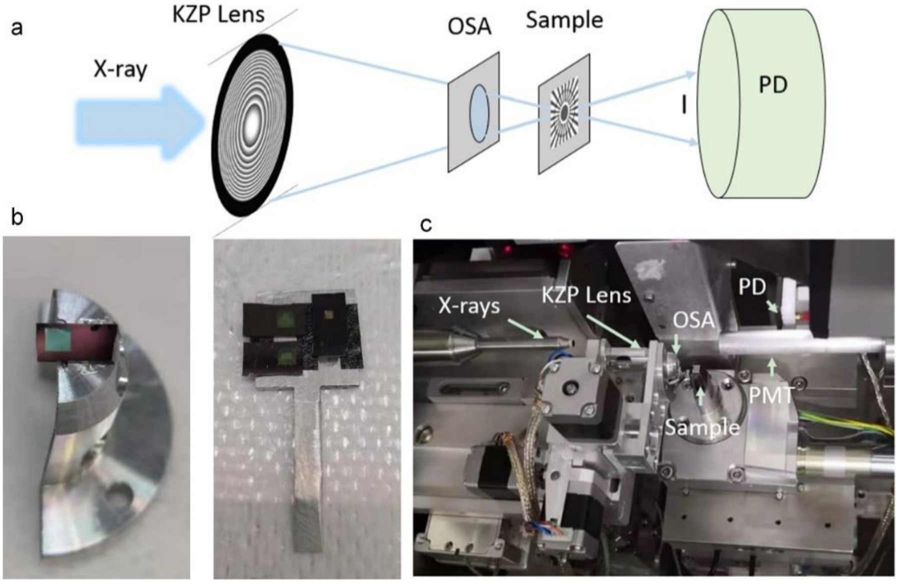

Figure S3 The optical-characterization setup used in this work. (a), the schematic diagram of the STXM, in which a set of slits acting as the source to produce coherent light illuminating the lens. The sample located in one of the focal planes, selected by an order sorting aperture (OSA), is raster scanned while the total transmitted light is measured by a point detector. (**b)**, Pictures of the lens and the Siemens Star prepared for the tests. (**c)**, The set-up for hard X-ray focusing tests.

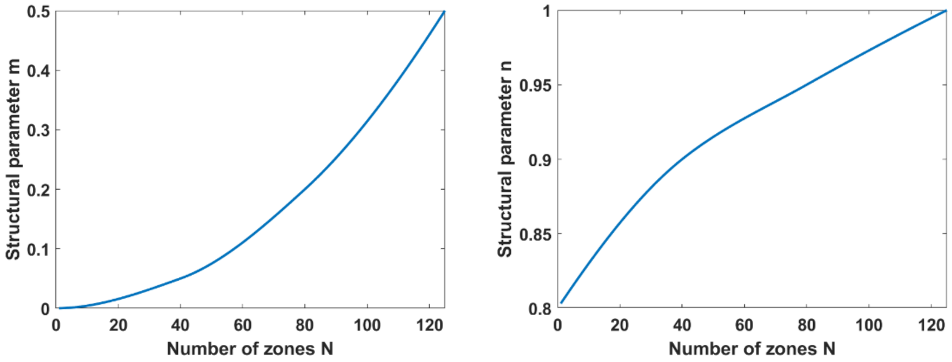

Figure S4 The change curves of structural parameters.

Table S1 Design parameters for lenses demonstrated in this work. The diameter of beam stop is 50 µm for all FZPs in this work

Lens	HSQ-KZP	Au-FZP	PMMA-KZP
Material	HSQ	Au	PMMA
Energy (eV)	500	500	500
Radius (µm)	50	50	50
Focal Length (mm)	4	4	4
Outmost zone width (nm)	200	200	200
Height (µm)	0.75	0.25	1
Exposure unit ring width (nm)	20	20	20

References

Henke, B. L., Gullikson, E. M. & Davis, J. C. (1993). *Atomic Data and Nuclear Data Tables*, 181-342.
